# Hyperpolarized ^83^Kr magnetic resonance imaging of alveolar degradation in a rat model of emphysema

**DOI:** 10.1098/rsif.2015.0192

**Published:** 2015-06-06

**Authors:** David M. L. Lilburn, Clémentine Lesbats, Joseph S. Six, Eric Dubuis, Liang Yew-Booth, Dominick E. Shaw, Maria G. Belvisi, Mark A. Birrell, Galina E. Pavlovskaya, Thomas Meersmann

**Affiliations:** 1 Sir Peter Mansfield Imaging Centre, Division for Respiratory Medicine, School of Medicine, University of Nottingham, Nottingham NG7 2RD, UK; 2 Respiratory Pharmacology, Pharmacology and Toxicology, Faculty of Medicine, National Heart and Lung Institute, Imperial College London, London SW7 2AZ, UK; 3 City Hospital Nottingham, Nottingham Respiratory Research Unit, Nottingham NG5 1PB, UK

**Keywords:** krypton-83, hyperpolarized noble gas MRI, pulmonary imaging, surface-sensitive contrast, animal model emphysema, nuclear electric quadrupolar relaxation

## Abstract

Hyperpolarized ^83^Kr surface quadrupolar relaxation (SQUARE) generates MRI contrast that was previously shown to correlate with surface-to-volume ratios in porous model surface systems. The underlying physics of SQUARE contrast is conceptually different from any other current MRI methodology as the method uses the nuclear electric properties of the spin *I* = 9/2 isotope ^83^Kr. To explore the usage of this non-radioactive isotope for pulmonary pathophysiology, MRI SQUARE contrast was acquired in excised rat lungs obtained from an elastase-induced model of emphysema. A significant ^83^Kr *T*_1_ relaxation time increase in the SQUARE contrast was found in the elastase-treated lungs compared with the baseline data from control lungs. The SQUARE contrast suggests a reduction in pulmonary surface-to-volume ratio in the emphysema model that was validated by histology. The finding supports usage of ^83^Kr SQUARE as a new biomarker for surface-to-volume ratio changes in emphysema.

## 1. Introduction

**Figure 1.**
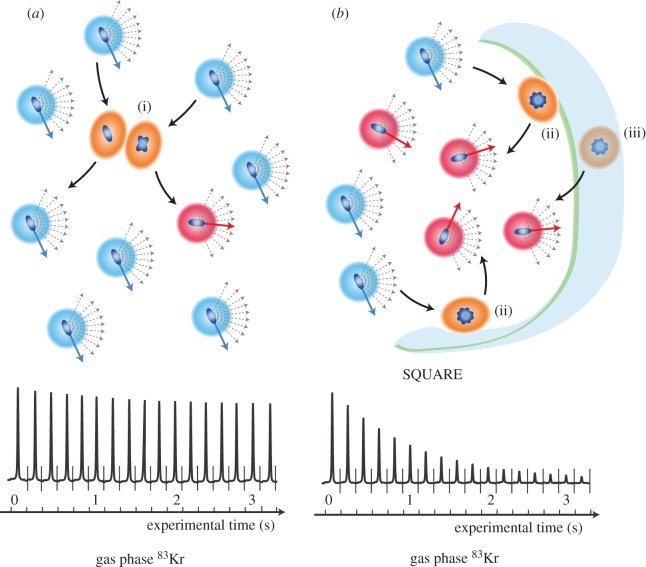
Illustration of the concept of hp ^83^Kr SQUARE contrast. The ^83^Kr atoms are depicted with the 10 possible spin states of this nuclear spin *I* = 9/2 isotope and with a non-spherical nucleus that possesses a nuclear electric quadrupolar moment. Hyperpolarized ^83^Kr atoms, drawn in blue, are depicted as occupying the lowest spin state (i.e. corresponding to the highest MRI signal intensity). However, longitudinal *T*_1_ relaxation will cause the spins to assume other states (atoms indicated in red), thus reducing the MRI signal intensity. Quadrupolar relaxation occurs when the electron cloud of ^83^Kr is forced out of the spherical shape. (*a*) In the bulk gas phase a non-spherical symmetry is caused by collisions (i) leading to the gas phase signal decay within minutes. (*b*) When surfaces are present, surface adsorption (ii) and possibly dissolution into deeper surface regions (iii) will lead to surface quadrupolar relaxation (SQUARE). Rapid exchange causes transfer of the SQUARE effect into the gas phase where the rapid signal decay is observed with typical *T*_1_ times approximately 1 s in rat lungs. See Material and methods section for further details.

Hyperpolarized krypton-83 (hp ^83^Kr) enables MRI contrast that is indicative of surface composition [1,2] and the surface-to-volume ratio (S/V) [3] in porous media. The *T*_1_-weighted MRI contrast is generated through surface quadrupolar relaxation (SQUARE) that causes S/V-dependent reduction in the hp ^83^Kr MR signal intensity as sketched in figure 1. Pulmonary SQUARE MRI contrast between major pulmonary airways and the alveolar regions has recently been demonstrated in excised rat lungs [4]. In this publication, the potential of hp ^83^Kr SQUARE contrast for lung pathophysiology is evaluated using *ex vivo* MRI of an established rat model of emphysema. The emphysema model was selected for this proof of concept study because of the well-known deterioration of the alveolar surface that is expected to cause increased SQUARE *T*_1_ times. Following the MRI acquisition, the alveolar surface deterioration can be quantified through histology.

Emphysema is a component of chronic obstructive pulmonary disease (COPD), the fourth leading cause of death worldwide [5]. The development of emphysema is mainly linked to cigarette smoking with a smaller proportion attributed to pollution, occupational exposure or intrinsic factors [6]. Over time, there is significant alveolar destruction with the resulting reduction in surface area for gas exchange with an accompanied loss of lung elasticity. Unfortunately, current routine investigations such as lung function tests often fail to diagnose the disease until the later stages [7] once a significant amount of damage has been done. There is therefore a need for new biomarkers to detect the disease in the early stages and to help separate COPD phenotypes [8].

Investigations of COPD and emphysema, in particular, have developed over the past decade with human studies using both computed tomography (CT) [9] and magnetic resonance imaging (MRI)-based techniques [10,11]. Hyperpolarized noble gas MRI [12–14] using ^3^He and ^129^Xe is able to provide measurements of ventilation and is able to delineate poorly ventilated and non-functioning lung regions [11,12]. Recently, visualization of delayed collateral ventilation into lung regions has been performed [15], providing information complementary to CT-based techniques. Furthermore, hp ^3^He and hp ^129^Xe provide measurements of the lung microstructure through the apparent diffusion coefficient (ADC) [16–24]. Using animal models of emphysema, the distinction between healthy and emphysematous tissue was possible through fractional ventilation generated hp ^3^He MRI contrast before an increase in alveolar diameter could be determined through histology [25,26]. Unlike fractional ventilation that is decreased in the disease model, ADC was found to be increased in long-term disease models developed over a six-month period [21]. A very promising new pulmonary MRI contrast can be obtained by probing the dissolved phase of hp ^129^Xe. A host of innovative new techniques, such as xenon polarization transfer contrast (XTC) [23,27–30], xenon alveolar capillary transfer (XACT) [31] and chemical shift saturation recovery spectroscopy (CSSR) [29,32,33] enable selective detection of gas phase xenon, tissue and plasma (TP) dissolved xenon and xenon interacting with red blood cells (RBC). For example, Dregely *et al.* [23] found a strong correlation between the XTC-based parameter (MXTC-F) and CT data. Using CSSR spectroscopy, Patz *et al.* [33] found a dramatic decrease in S/V in emphysema patients, while alveolar septal thickness and capillary transit time was not affected. In general, the findings suggest that a sensitive probe for S/V changes is the key to early emphysema diagnosis.

This work sets forth the verification of a new type of hyperpolarized noble gas modality for pulmonary studies, i.e. hp ^83^Kr SQUARE MRI contrast [4], which may provide a significant addition to existing methodology. Previous work exploring the underlying conceptual physics found SQUARE to be the dominant cause of ^83^Kr *T*_1_ relaxation observed in the gas phase in high S/V porous media such as lungs. See figure 1 and Material and methods section for an explanation of the SQUARE concept. The purpose of this work is to determine whether the SQUARE effect is sensitive enough to serve as a probe for disease-related lung physiological changes.

The potential significance of SQUARE MRI contrast is that it may enable a novel type of biomarker for pulmonary pathophysiology through a fundamentally different physical effect compared with those used in other pulmonary diagnostic techniques such as hp ^3^He ADC and hp ^129^Xe dissolved phase measurements. For example, the associated timescale of the ‘surface probing’ is 1–2 orders of magnitude longer than that of ADC measurements. The nature of the contrast generation may provide a methodology sensitive not only to S/V but also to the chemical composition of the surface. Furthermore, in model surfaces quadrupolar noble gas (i.e. ^131^Xe) relaxation was affected by microscopic surface fine structure [34,35]. Similarly, ^83^Kr SQUARE is likely to be sensitive to surface corrugation and may, therefore, provide different S/V values compared with dissolved xenon-based techniques [33].

Informed by the surface studies of previous models [1–3,36,37], the underlying hypothesis of this publication is that hp ^83^Kr SQUARE MRI contrast can serve as a biomarker for the alveolar S/V reduction caused by emphysema. Confirmation of the S/V hypothesis in a preclinical small animal model is a crucial milestone for the development of hp ^83^Kr MRI. The intratracheal elastase exposure of rat lungs to porcine pancreatic elastase (PPE) generates an established model of emphysema whereby initial inflammation is produced with the subsequent development of airspace enlargement and destruction [38–41]. The experimentally and regulatory less demanding *ex vivo* set-up [14,42] was used for the MRI measurements in this proof of concept work to set the basis for future *in vivo* preclinical and clinical studies.

## 2. Material and methods

### 2.1. SQUARE contrast

Figure 1 illustrates the mechanism of SQUARE with the nuclear spin *I* = 9/2 isotope ^83^Kr. Atomic nuclei are positively charged electric monopoles, however, the nucleus of any spin *I* > 1/2 isotopes is non-spherical leading to a non-uniform nuclear electric charge distribution. The resulting nuclear electric quadrupole moment can interact with the surrounding electrons if the noble gas atom is ‘distorted’—i.e. if the electronic cloud assumes a non-spherical symmetry. In the bulk gas phase, far away from surfaces (figure 1*a*), collisions events with other atoms cause rapidly fluctuating electron cloud distortions that results in ^83^Kr quadrupolar relaxation with *T*_1_ times of several minutes at ambient pressure. SQUARE (figure 1*b*) occurs when surface adsorption and possibly dissolution into deeper surface regions takes place. SQUARE can typically not be observed directly owing to strong line broadening and the limited number of atoms at the surface at any given time. However, the SQUARE effect is transferred into the gas phase through rapid exchange and, depending on the S/V ratio, can tremendously accelerate the decay of the hp gas phase signal. SQUARE contrast is, therefore, potentially sensitive to S/V, surface composition and surface temperature. SQUARE caused by high S/V alveolar region of rat lungs reduces the gas phase T_1_ time to approximately 1 s leading to the rapid hp ^83^Kr signal decay in the series of small flip angle spectra in figure 1*b*.

### 2.2. ^83^Kr Spin exchange optical pumping, compression and transfer

Hyperpolarized ^83^Kr was produced in batch mode by SEOP as described previously described in detail [43]. All MRI was performed using enriched ^83^Kr (99.925% ^83^Kr, CHEMGAS, Boulogne, France) to improve the available signal intensity. A 15% krypton 85% N_2_ (99.999% purity, Air Liquide, Coleshill, UK) mixture was used to reduce the consumption of expensive isotopically enriched ^83^Kr. SEOP build-up times of 12 min, corresponding to greater than 92% of the steady-state polarization were used to reduce the experimental duration. The hyperpolarized gas extraction unit described previously [4,44] was used to allow for below ambient pressure SEOP [4,43] performed at 55–65 kPa [43]. Overall, the method produced a ^83^Kr nuclear spin polarization of *P* = 16–17% after accounting for depolarization occurring in the gas extraction process [44]. An approximate volume of 12–16 ml of the hp gas mixture (1 : 6.7 Kr:N_2_) was obtained for lung imaging every 12 min. Since no viable method currently exist to separate hp ^83^Kr from the mixture, it is instructional to report the apparent spin polarization of approximately *P*_app_ = 2.5%. The apparent polarization is the nuclear spin polarization *P* times the fraction of krypton in the hp gas mixture [43]. A discussion of the nuclear spin polarization *P* for isotopes with nuclear spin *I* > 1/2 can be found in [45].

### 2.3. Hyperpolarized gas inhalation

The lungs were suspended in a 5% glucose solution (weight/volume) in the ventilation chamber as described in previous work [14,42]. The chamber was then placed in the centre of the superconducting magnet bore with the temperature kept constant at 295 K throughout the experiments. Active inflation of the lung was accomplished by pulling to a ventilation syringe volume (*V*_s_) of 8 ml. Corresponding inhaled volumes (*V*_i_) were measured separately using the water displacement technique on gas exhalation [14] and are shown in table 1. To limit gas trapping (particularly notable in the PPE-treated lungs) the *ex vivo* lungs were deflated over 30–60 s from *V*_s_ = 8 ml to maximum exhalation (*V*_s_ = 0 ml) as has been reported elsewhere [46,47] before hp ^83^Kr inhalation.

**Table 1.** Demographic data from satellite subjects (histology only) and those used for hp ^83^Kr imaging (with subsequent histology). Summary of rat weights, whole lung mean alveolar area ± standard deviation of the mean, inhaled volumes (*V*_i_) ± standard deviation corresponding to inflation (syringe) volume *V*_s_ = 8 ml with associated inhalation pressures ± standard deviation. No values for *V*_i_ were determined in the histology groups. Values omitted were not measured.

				lung usage	
	identifier	rat weight (g)	whole lung MAA (10^4^ μm^2^)	*MRI*: hp ^83^Kr MRI including histology, *Histology*: (satellite group)	inhaled volume in MRI, *V*_i_ (ml)
control	CL.1	492	—	MRI	7.0 ± 0.3
	CL.2	555	2.5 ± 0.1	MRI	6.8 ± 0.1
	CL.3	499	3.7 ± 0.6	MRI	6.8 ± 0.1
	CL.4	400	1.9 ± 0.1	histology	N/A
	CL.5	412	2.5 ± 0.4	histology	N/A
elastase (PPE) treated	EL.1	390	6.3 ± 1.1	MRI	5.9 ± 0.7
	EL.2	508	6.8 ± 1.1	MRI	6.1 ± 0.6
	EL.3	416	5.1 ± 1.0	MRI	6.9 ± 0.3
	EL.4	440	4.5 ± 0.6	MRI	7.3 ± 0.4
	EL.5	513	3.6 ± 1.0	MRI	6.1 ± 0.2
	EL.6	382	4.8 ± 1.4	histology	N/A
	EL.7	436	10.8 ± 6.2	histology	N/A

### 2.4. Magnetic resonance imaging protocol

MRI experiments were performed using a vertical bore 9.4 T Bruker Avance III microimaging system (Bruker Corporation, Billerica, MA, USA) with a standard Bruker double saddle coil tuned to ^83^Kr resonance frequency of 15.40 MHz. The internal diameter (ID) of the coil was 30 mm.

Coronal images were acquired into 64 × 32 matrices using a variable flip angle (VFA) FLASH protocol (TE = 1.8 ms, TR = 12.6 ms) [48]. Rectangular RF pulses of constant duration of 0.3 ms and variable power levels were used in all experiments. The imaging protocol had a total acquisition time 0.405 s to minimize the effects of unwanted *T*_1_ decay during acquisition. To obtain *T*_1_-weighted images [4], each imaging sequence was started after a programmed time delay, *τ*_d_, post inhalation. Typically, *τ*_d_ = 0.2 s, 0.7 s, 1.2 s, 1.7 s and 2.2 s were used, although some of the *T*_1_ maps were calculated from a series of four images with *τ*_d_ = 0.5 s, 1.0 s, 1.5 s and 2.0 s. The inhalation itself was accomplished manually by reducing the pressure in the artificial pleural cavity using the ventilation syringe [14,42]. Slight alternations in the timing of the manual inhalation procedure (approx.±0.2 s) were deemed acceptable. In all imaging experiments each individual image was acquired from a single inhalation cycle using one VFA FLASH acquisition and no signal averaging. The resulting FOV was 50.9 × 40.7 mm^2^.

### 2.5. Image reconstruction and *T*_1_ analysis

The raw 32 × 32 datasets were apodized using sine-bell squared function and zero filled to 64 points in each spatial domain before Fourier transformation. Final image resolution was 0.795 × 0.635 mm^2^ in the frequency encoding (longitudinal) and in the phase encoding (transverse) directions, respectively. These final 64 × 64 magnitude images were exported to IGOR Pro (v. 6.01, Wavemetrics, Lake Oswego, OR, USA) for *T*_1_ analysis.

The *T*_1_ datasets were created for each series of images by combining the images acquired at the individual time delays *τ*_d_ into a three-dimensional dataset where the first image in the set corresponded to the image acquired at the smallest time delay, *τ*_d_ as described in [4]. The *T*_1_ values outside of the 0 s < *T*_1_ ≤ 6 s range were rejected as physically not meaningful. The *T*_1_ values within the range of 0 s < *T*_1_ ≤ 6 s but located outside the lung contour region, or the region of interest (ROI), were also discarded. The ROI was determined from the first image in each *T*_1_ set. These final *T*_1_ maps were used to produce *T*_1_ histograms for each lung reported in this study.

The *T*_1_ data collected from the ROI in each *T*_1_ map were binned into 200 intervals with 0.03 s increment. The resulting histograms were analysed using build-in Multipeak 1.4 fitting procedure and automated peak picking. A bi-modal Gaussian distribution of *T*_1_ values in the lung was assumed because of the distinct alveolar and conducting airway compartments present in the lung. The results of the analysis returned the most probable (expected value, EV) relaxation time, 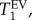 its probability and distribution measured as full width, half maximum, FWHM(*T*_1_) for each of the two Gaussian components.

### 2.6. Model characterization: the elastase model and preparation for *ex vivo* MRI

Male Sprague-Dawley rats (260–300 g) were purchased from Harlan UK Ltd. (Bicester, UK). Home Office guidelines for animal welfare based on the Animals (Scientific Procedures) Act 1986 were strictly observed. Experimental emphysema was induced by instilling one dose of 120 U kg^−1^ PPE (Merck Chemicals Ltd, Nottingham, UK) intratracheally at 1 ml kg^−1^ directly into the airways under general anaesthesia (inhaled isofluorane 4% with medical grade oxygen for 3–4 min) [40,49]. Control animals were similarly treated with 1 ml kg^−1^ sterile saline (Fresenius Kabi Ltd, Manor Park, UK) at the same time. At 28 days after intratracheal dosing animals were weighed and euthanized by overdose of sodium pentobarbital 200 mg kg^−1^ intra-peritoneal (Merial Animal Health, Harlow, UK). After confirmation of death, a catheter was inserted into the caudal vena cava to allow flushing of the pulmonary circulation with 20 ml heparin 100 IU ml^−1^ (Wockhardt UK Ltd, Wrexham, UK) in 0.9% saline solution (Baxter Healthcare Ltd, Thetford, UK) followed by 20 ml^−1^ Dublecco's phosphate buffer solution (D-PBS, Sigma-Aldrich Ltd, Gillingham, UK) to remove residual blood from the pulmonary circulation. The heart and lungs were subsequently removed *en masse*.

Lungs for *ex vivo* hp ^83^Kr imaging had a polytetrafluorethylene (PTFE) adapter tube inserted into the trachea 5–10 mm above the carina and sutured into place. The heart and lungs were then suspended in 5% glucose solution (weight/volume) (Baxter Healthcare Ltd, Thetford, UK) with the trachea pointing downwards as has previously reported [14,42]. The *ex vivo* lungs were repeatedly inflated with 8–10 ml of room air to check for gas leaks either from the suture around the trachea or the lungs themselves. The lungs were chilled to 278 K for transportation to the imaging facility during which time they were repeatedly inflated with 8–10 ml of air at 30–60 min intervals. Time from lung harvest to the start of imaging was no more than 8 h for each lung.

### 2.7. Model characterization: alveolar cross-section measurements

PPE-induced changes in air space were measured in both a satellite group of rats and on the *ex vivo* lungs used for hp ^83^Kr MRI. Lungs from both groups were similarly prepared by inflating to forced vital capacity (25 cm H_2_O) with 5% formalin–saline solution (Sigma-Aldrich Ltd, Gillingham, UK). The trachea was then tied off and the lungs and heart removed *en bloc* and placed in formalin. After at least 24 h in formalin, an experienced histologist processed the lungs. Sections were cut from the wax embedded samples using a microtome and stained with H&E.

Experimental emphysema was assessed by measuring average air space area using a method describe by Belloni *et al.* [50] and used previously [40]. Briefly, computer-assisted image analysis was performed using an Olympus BX40 microscope and Zeiss image-processing software (Imaging Associates, Bicester, UK). Using colour thresholding techniques, the total alveolar air space area and the number of air spaces was measured from five random fields per sample. From these figures the mean air space area was calculated. Any fields containing airways or vasculature were excluded. The person assessing the slides was blinded to the treatment. Details of control and PPE-treated lungs are shown in table 1 with average measurements of mean alveolar area (MAA) in the satellite group and in those lungs processed post *ex vivo* hp ^83^Kr MRI.

## 3. Results

### 3.1. Comparison hyperpolarized ^83^Kr magnetic resonance images and resultant *T*_1_ maps between control and porcine pancreatic elastase-treated groups

**Figure 2.**
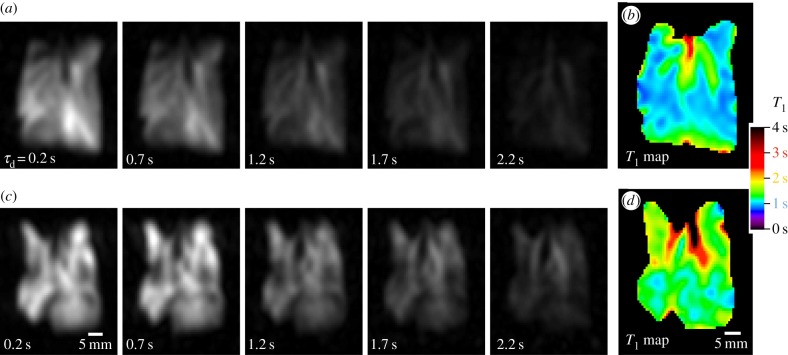
Series of hp ^83^Kr MR images with resultant *T*_1_ maps in control and PPE-treated lungs. Each image was acquired using a new delivery of hp ^83^Kr. (*a*) VFA FLASH MRI with no slice selection in control lung CL.2, using a variable relaxation delay, *τ*_d_, ranging from 0.2 to 2.2 s between hp gas inhalation and acquisition as indicated in the figure. Note that the major airways are less affected than the lung parenchyma by increasing *τ*_d_ values. (*b*) The resultant SQUARE *T*_1_ map for the control lung displays longer *T*_1_ values (green) for the major airways and shorter values for the alveolar region (blue). (*c*) VFA FLASH MRI as in (*a*) for the PPE-treated lung EL.1. The lung exhibits heterogeneity in ventilation that is likely caused by the PPE treatment but is not a clear indicator for emphysema. (*d*) The resultant SQUARE *T*_1_ map of the PPE-treated lung displays prolonged relaxation times (green) in the alveolar area compared with the control SQUARE *T*_1_ map above.

**Figure 3.**
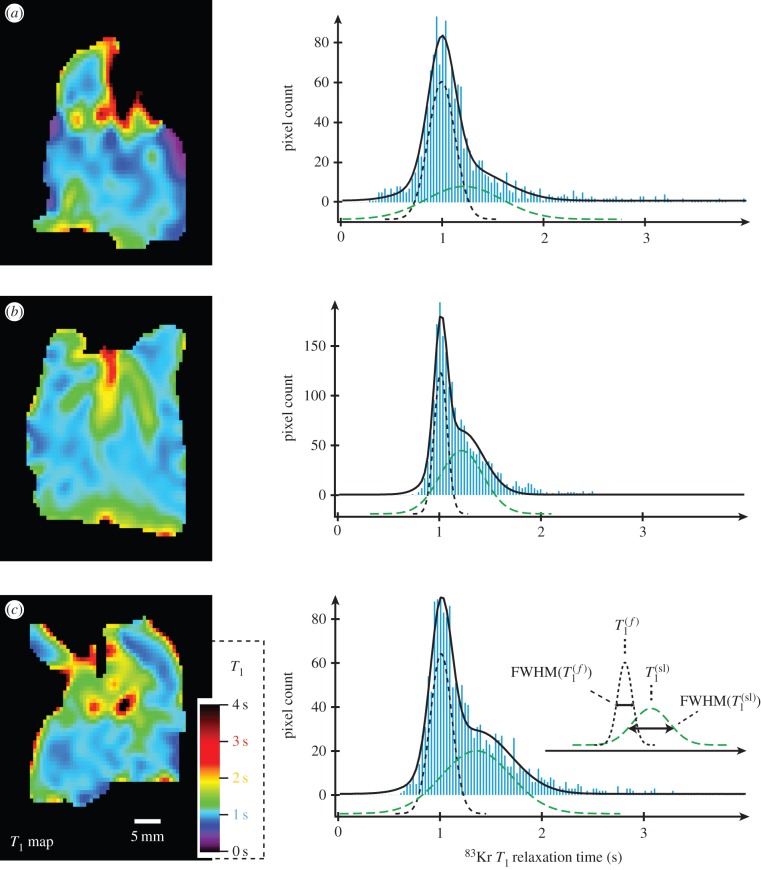
^83^Kr MRI *T*_1_ maps (SQUARE contrast) of three control lungs and their corresponding histograms. The SQUARE *T*_1_ maps have been obtained as described in figure 2. Blue colours in the alveolar regions indicate short *T*_1_ values around 1 s. The histograms depict the frequencies (i.e. pixel count from the SQUARE *T*_1_ maps) of *T*_1_ values within 0.03 s intervals. Bimodal fitting leads to a narrow distribution of fast relaxing pixels centred around 
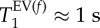
 (black dotted line—displayed with negative pixel count offset for clarity) and a broader distribution centred around 
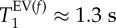
 (green dashed line -negative pixel count axis offset). The solid black line displays the sum of the two Gaussian distributions. The characteristic *T*_1_ times are explained in insert in histogram (*c*) and the specific values for each lung and averaged data are listed in table 2 with lung CL.1 shown in (*a*), CL.2 in (*b*) and CL.3 in (*c*).

Examples of hp ^83^Kr MR images acquired using the series of increasing delay times, *τ*_d_, and the resultant SQUARE *T*_1_ maps are displayed in figure 2. Compared with the control lungs, a significant heterogeneity of ventilation was found for in the PPE-treated lung. However, heterogeneous ventilation is not particular to emphysema as it can be caused by a variety of diseases and effects. For example, heterogeneous ventilation was also observed in two of the *ex vivo* control lungs shown in figure 3. Furthermore, ventilation MRI with a better image resolution can be obtained through hp ^3^He or hp ^129^Xe and the focus of this work was on *T*_1_ relaxation generated contrast. The hp ^83^Kr MR images in figure 2*a* demonstrate how increasing *τ_d_* delay time between inhalation and image acquisition (i.e. from 0.2 s to 2.2 s) leads to a decrease in hp ^83^Kr signal intensity due to *T*_1_ relaxation. As sketched in figure 1*a*, the ^83^Kr gas phase relaxation outside the lung is in the order of minutes [51]. The fast ^83^Kr *T*_1_ relaxation found within the lung is therefore predominantly caused by interactions of the krypton atoms with the surrounding surface—i.e. by the SQUARE effect. Note ‘surface’ does not necessarily refer solely to the outermost surfactant layer and deeper levels, including cell membranes, may contribute as long as fast exchange transfers the depolarized ^83^Kr back into the alveolar gas phase where MRI signal detection takes place (only gas phase ^83^Kr signals are MR observable—see also figure 1). In any case however, the ^83^Kr relaxation time is expected to be sensitive to the S/V ratio and the purpose of this study is to investigate whether SQUARE can serve as an indicator for the emphysema model.

Although other factors, such as chemical composition affect SQUARE [3] the expected strong dependence on S/V ratios is the likely cause for regions with lower S/V, such as the major airways in the control lung, to experience a slower *T*_1_ relaxation and thus a slower depolarization rate. These areas remain ‘bright’ in the MR images while areas with higher S/V lead to ‘dark’ regions of faster depolarization. The *T*_1_ map calculated from the delay time *τ_d_*-dependent signal decay leads to the actual SQUARE contrast images shown in figure 2*b* for the control lung and figure 2*d* for the elastase-treated lung. Prolonged relaxation times were found for the major airways compared with the alveolar region in the control lung in figure 2*b* (see also ref. [4]). PPE-treated lungs, serving as a model for emphysema, are imaged for the first time with hp ^83^Kr in this work. The hp ^83^Kr SQUARE *T_1_* map of the lung shown in figure 2*d* exhibits increased *T*_1_ values for the alveolar region compared with control lungs. This is reflected in the SQUARE *T*_1_ map that displays an elevated level of green colour compared with figure 2*b* (far right).

Figure 3 depicts the *T*_1_ maps of all three control lungs. The data from the *T*_1_ maps were used for histogram analysis with frequencies of *T*_1_ values within 0.03 s intervals. The *T*_1_ relaxation time distribution of the histograms was analysed using bimodal Gaussian distribution function. The four characteristic *T*_1_ times from this fitting—i.e. the most probable (expected value, EV) relaxation time, 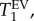 of each of the two Gaussian components and their distribution measured as full width, half maximum, FWHM(*T*_1_), are listed in table 2. The Gaussian distribution of the fast relaxing component centred around 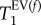 is indicated by a black dotted line in the histograms, whereas the slow relaxing group, centred around 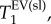 is displayed by the green dashed Gaussian curve. The sum of both components results in the black solid line that very closely resembles the histograms, suggesting that bimodal fitting is a good approach for extraction of parameters that are characteristic for the SQUARE behaviour of a lung.

**Table 2.** Characteristic *T*_1_ times from bimodal fitting of the histograms of all lungs used in this work.

		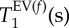	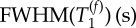	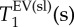	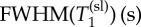
	rat identifier	mean	mean	mean	mean
control lung	CL.1	0.9958	0.19619	1.2353	0.53927
	CL.2	1.0130	0.091234	1.2189	0.30050
	CL.3	1.0099	0.14987	1.3556	0.48057
average ± standard deviation		1.00620 ± 0.009	0.14576 ± 0.053	1.2699 ± 0.075	0.44011 ± 0.124
combined histogram of control lungs (figure 5, blue histogram)		1.0112	0.13073	1.2494	0.38797
elastase (PPE) treated lung	EL.1	1.2559	0.12770	1.4787	0.32787
	EL.2	1.2311	0.30498	1.7067	0.52674
	EL.3	1.3697	0.28202	2.0474	0.63887
	EL.4	1.1576	0.21975	1.5708	0.71203
average ± standard deviation		1.25358 ± 0.088	0.23361 ± 0.079	1.7009 ± 0.249	0.55138 ± 0.167
combined histogram of elastase (PPE) treated lungs (figure 5, red histogram)		1.2734	0.28201	1.7288	0.54928
	EL.5	0.94994	0.1333	1.2257	0.4500

**Figure 4.**
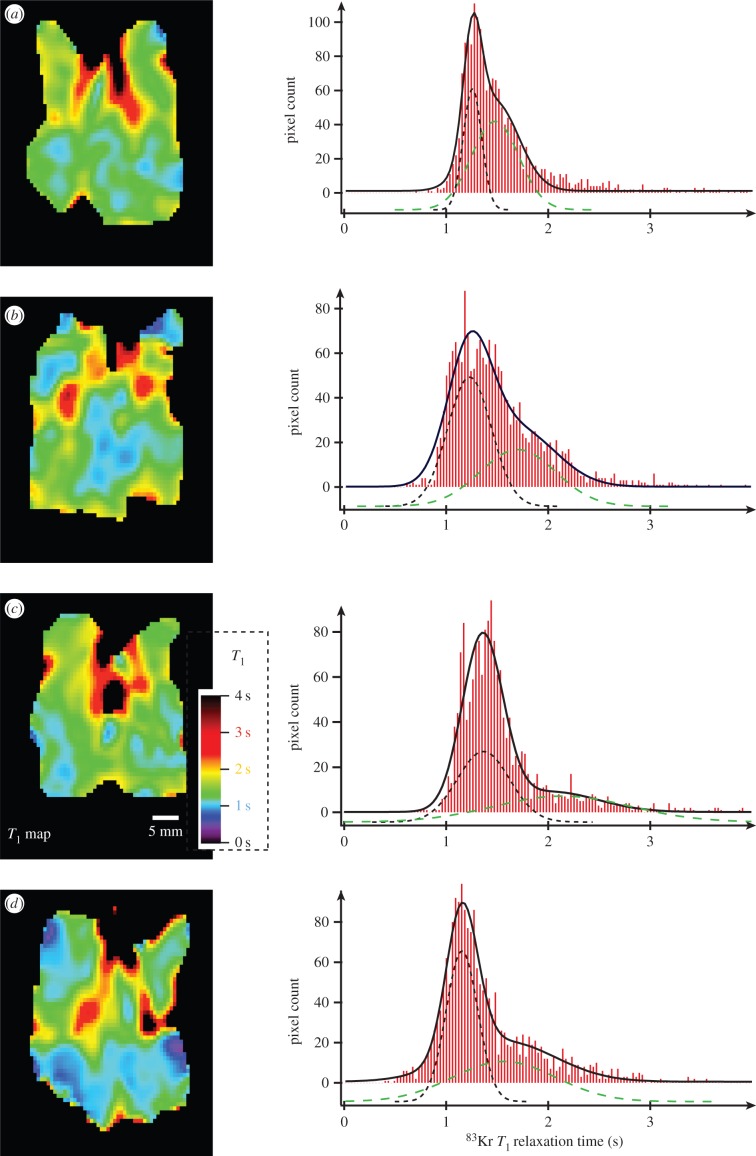
^83^Kr MRI *T_1_* maps (SQUARE contrast) of four of five PPE-treated lungs and their corresponding histograms. Green colours have now become more prevalent in the alveolar regions indicating increased *T*_1_ values compared with those shown in figure 3. The histograms display the shift to larger *T_1_* values with 
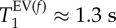
 (black dotted line), 
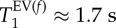
 (green dashed line) with exact data listed in table 2. (*a*) Lung EL.1, (*b*) EL.2, (*c*) EL.3 and (*d*) EL.4. As in figure 3, the fast relaxing group has a narrower distribution than the slower relaxing group.

**Figure 5.**
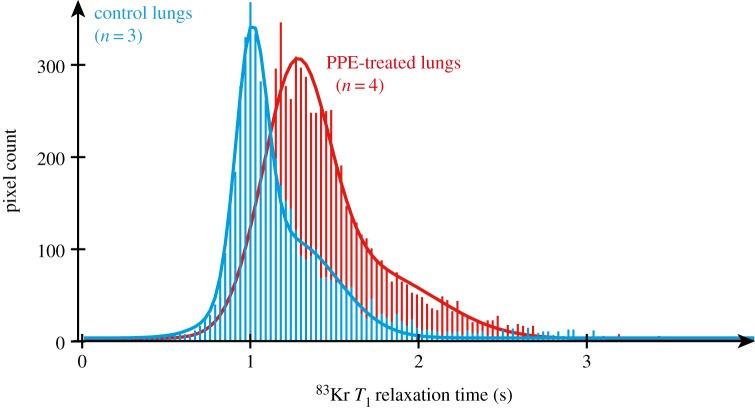
Histogram of all added *T_1_* frequencies. Data were obtained from the three control lungs (blue) in figures 3 and 4 elastase (PPE—red) treated animals (figure 4). The characteristic *T*_1_ data from bimodal fitting (as in figures 3 and 4) are listed in table 2 and are very similar to the averaged characteristic *T*_1_ values for control and elastase group. The sum of the bimodal fitting resulted to the blue solid line for the control group and the red solid line for the PPE group.

Figure 4 shows the SQUARE *T*_1_ maps and histograms for PPE-treated lungs. SQUARE *T*_1_ maps and histograms display marked differences between PPE-treated and control lungs as can also be noted by the characteristic *T*_1_ times from the bimodal fitting listed in table 2. The combined histograms for all three control lungs are shown together with the combined histograms of the four elastase-treated lungs in figure 5.

**Figure 6.**
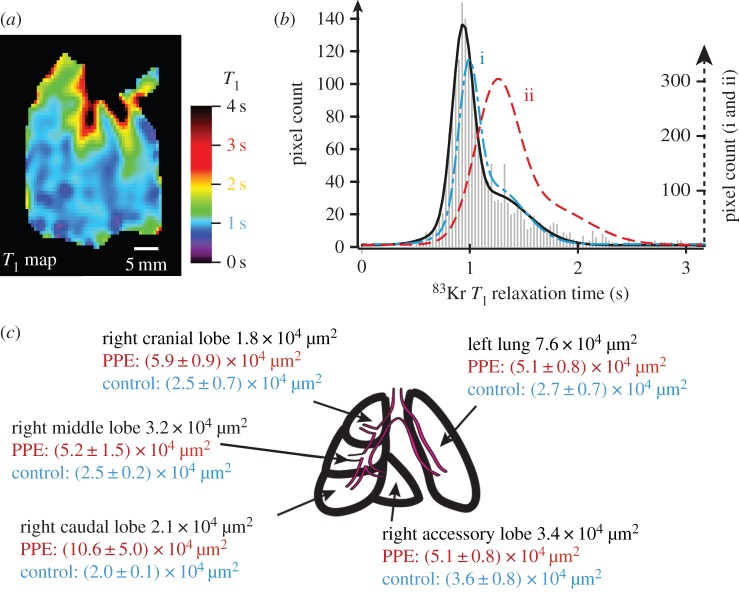
^83^Kr MRI *T*_1_ maps (SQUARE contrast) of the PPE-treated lung EL.5 (*a*) and its corresponding histogram (*b*) with comparison of lobar mean alveolar area values (*c*). The grey bars indicate the experimental frequency of *T*_1_ values and the solid black line is the combined result from bimodal fitting (the two individual components not shown but characteristic times listed in table 2). For comparison the outline of the resulting histogram from figure 5 is shown for the control group (blue dash–dotted line—i) and the elastase group (red dashed line—ii). Both the appearance of the SQUARE contrast and the histogram demonstrate that this lung has similar ^83^Kr *T*_1_ values to those lungs in the control group. (*c*) The mean alveolar area from lobar histology of PPE-treated lung EL.5 after the MRI experiments (black numbers) suggest that the emphysema model did not develop in all but the left lobe when compared with the mean values for the control (blue text—average from four control lungs) and PPE-treated groups (red text—average from six PPE lungs).

Figure 6 displays a fifth lung (EL.5) from the PPE group that exhibits SQUARE characteristics very similar to that of control lungs. However, table 1 shows that the averaged MAA is fairly low with MAA = 3.6 × 10^4^ µm and thus is similar to that of the control group. The regional (lobar) MAA values are shown in figure 6*c* together with the averaged MAA values from the control and elastase groups. All lobes except for the left lung lobe of the lung display values in line with the control group. The left lung lobe exhibits increased MAA significantly above the average from the PPE group.

## 4. Discussion

**Figure 7.**
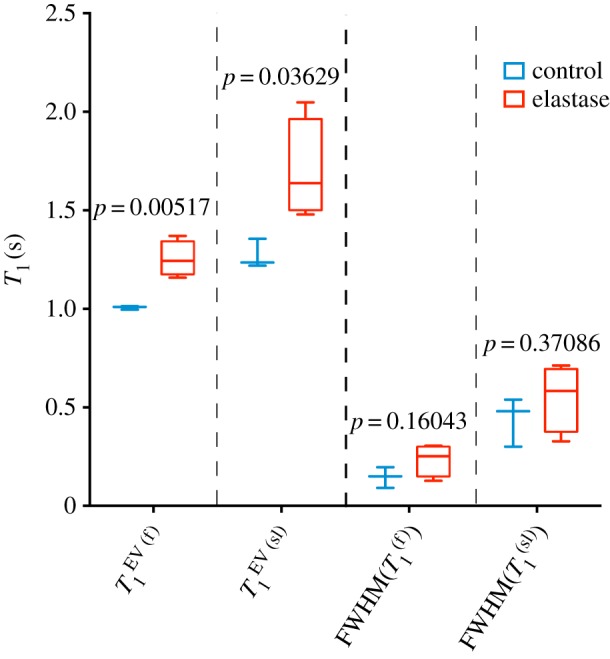
Box diagram of the characteristic data listed in table 2. The MRI 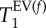 data between control and elastase (PPE) group do not overlap. The same observation is made for the 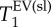 data. In contrast, the distribution of *T*_1_ values for the two modes of a histogram (characterized by FWHM(*T*_1_)) significantly overlaps between control and PPE group, indicating that no significant statistical difference to be expected from the *T*_1_ spread. However, the variation in 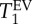
_,_ in particular 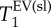, between the individual animals is more pronounced in the PPE group than in the control animals as visible in the added data in figure 5.

Two of the control lungs in figure 3 display clear ventilation heterogeneity as parts of the lungs remain dark in the hp ^83^Kr MR images. The cause of the heterogeneity is unknown but the lungs were transported over a 3-h period, subsequent to excision, between the location of animal holding facility at Imperial College and the hp MRI facility at Nottingham. Nevertheless, the analysis of the SQUARE *T*_1_ maps and histograms of all three control lungs led to comparable results with similar bimodal *T*_1_ distribution. Figure 5 depicts the combined *T*_1_ frequencies for the control group and for the elastase-treated animal lungs. The characteristic *T*_1_ values from bimodal fitting are listed in table 2 and show little variation within the two groups (i.e. control and PPE group). The pronounced increase in the 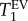 times for the PPE group in figure 5 and table 2 shows that the 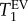 data can serve as an indicator for the development of the symptoms in the emphysema model. Presumably, the 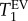 data are correlated to MAA. This correlation is expected from previous work with model surfaces but requires further corroboration in future studies. In any case however, the FWHM(*T*_1_) data are not associated with the MAA differences between the two groups. This view is further supported by the box diagrams in figure 7, which show no overlap in 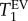 data between the two groups. However, the associated FWHM(*T*_1_) data almost completely overlap and are therefore unlikely to be useful as a biomarker for changes in MAA associated with the disease model.

Figure 7 also provides the results of statistical significance testing using Student's *t*-test at a critical significance level of *α* = 0.05. The null hypothesis, i.e. assuming no statistically significant difference in 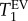 values between control and PPE group, was rejected for 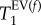 and 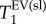 but not for 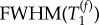 and 
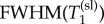
. This suggests that two parameters, namely 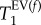 and 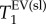, are useful biomarkers for the elastase model of emphysema and may be sensitive to the increase in MAA.

A correlation between MAA and SQUARE would be caused by the dependence on lung S/V ratio. A decreasing S/V is expected from increasing MAA in the emphysema model [21,26] and it is known from previous work with model surfaces [3] that *T*_1_ ∝ V/S. In this work, the *T*_1_ increase was not quantitatively correlated with the increase in MAA but a statistically significant increase in 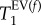 and 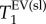 was observed for the emphysema model with increased MAA in histology compared with the control group. The bimodal appearance of the histogram could be caused by the presence of high S/V in the alveolar region and lower S/V within the airways. The alveoli may cause the fast relaxing mode described by 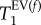 with a narrow 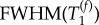 as the alveolar region will be fairly homogeneous. The airways, on the other hand, may contribute to the slow 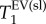 mode with a broad distribution and hence a larger 
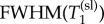
 due to the higher variability in S/V within the various airway generations. Note the relative contribution (in pixel count) between the two modes may not be quantitative due to the different extent of depolarization during the initial 0.2 s (fixed) delay and image acquisition. If the slow mode is indeed (at least partially) caused by the airways, the increase in 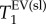 in the elastase-treated rat lungs would suggest that the S/V in the airways is affected by the disease model. Although this point was not further investigated, nitrogen chaser hp ^83^Kr MRI could be used for future exploration where the airways are largely purged with a small volume of N_2_ at the end of the inhalation [42].

The fifth lung (EL.5) from the PPE-treated group was excluded from the statistical analysis because the MAA increase developed asymmetrically in the left lung lobe only, possibly owing to localized elastase deposition. From the data in figure 6*c* one would expect *T*_1_ values similar to those from control lungs in all lobes except for the left lung lobe that exhibited very high MAA values. The SQUARE *T*_1_ map and the corresponding histogram are indeed very similar to that of the control lungs, however, this also includes the *T*_1_ values found in the left lobe. One would expect that the unexposed part of the lung to shows control lung behaviour, as indeed observed, but one would also expect very long *T*_1_ times from the left lobe. However, the left lobe may have been damaged too excessively and some regions may, therefore, no longer be ventilated. These areas will not be able to contribute to the SQUARE *T*_1_ map. Although non-ventilated areas should appear as ‘dark’ regions in the hp ^83^Kr MR images, these zones may be masked by MR signals from unaffected regions that contribute to the non-slice selective images.

For this work, excised lungs have been used. Two of the control lungs exhibited ventilation defects that may have been caused by the excision procedure and long transport period between the laboratories. Although this situation is non-ideal, the experiments demonstrate that the SQUARE *T*_1_ contrast was little affected by any damage arising from lung excision, *ex vivo* transport, and *ex vivo* experiments. The MAA, obtained after MR image acquisition, were in the expected range for PPE and control group, except for EL.5 as discussed above.

This work demonstrated that hp ^83^Kr SQUARE can serve as a biomarker for the elastase model of emphysema, in all likelihood because SQUARE senses changes in S/V. If the absence of harmful X-ray radiation is a strong motivation to explore the various pulmonary MRI techniques, a further potential advantage arises with hp ^83^Kr: the SQUARE effect can be used as a pulmonary biomarker without the presence of pulsed magnetic field gradients (PFGs). Unlike ADC measurements where PFGs are required to generate the MRI contrast, SQUARE is solely caused by a relaxation rooted physical effect. Although PFGs are still needed for the MR image generation, hp ^83^Kr SQUARE may have a potential as a global lung surface biomarker for pulmonary screening without spatial resolution. Since the effect of relaxation can be measured remotely [37], SQUARE may be observed without an MRI scanner if the effect is remotely detected upon exhalation into a small bench-top NMR device.

The study presented here was focused on providing a first demonstration of hp ^83^Kr SQUARE usage as an MRI contrast and biomarker for pulmonary pathophysiology in an animal model. Remarkably, using completely different physical concepts from those described here, krypton gas usage has been previously reported for contrast generation in dual energy computed tomography (DECT) [52]. The high-volume inhalation of a mixture of 80% krypton and 20% O_2_ did not lead to adverse effects in COPD patients. Compared with CT, SQUARE MRI is expected to require lower krypton quantities for usable contrast generation. Finally, molecular oxygen does not dramatically accelerate ^83^Kr T_1_ relaxation and SQUARE measurements in rodent lungs [42] were unaffected by the presence of up to 40% O_2_.

## 5. Conclusion

Informed by the previous S/V dependence of SQUARE in model surfaces, the aim of this work was to demonstrate the ability of hp ^83^Kr SQUARE MRI to serve as a biomarker for the elastase model of emphysema. It was demonstrated that two characteristic 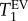 times, obtained from bimodal fitting of the histograms, enable statistically significant distinction between emphysema model and control lung. Beyond statistics, the difference between the control group and emphysema model can also be identified from visual inspection of the ^83^Kr SQUARE images shown in figures 3 and 4. The use of the simpler *ex vivo* model has allowed for rapid confirmation of the imaging technique despite some ventilation defects in the control group. Hp ^83^Kr SQUARE may serve as a potential biomarker for pulmonary disease-related S/V ratio changes. Future quantification of the effect and comparison with other hp noble gas modalities will provide further evaluation of this technique. Because neither the application of magnetic field gradients nor the detection within high magnetic fields is required for SQUARE measurements, this new biomarker should also be explored for potential pulmonary mass screening using small bench-top devices.
